# Supplementary material for: Spatiotemporal dynamics of grassland aboveground biomass in northern China and the alpine region: Impacts of climate change and human activities
Source: PLoS One. 2024 Dec 16;19(12):e0315329. doi: 10.1371/journal.pone.0315329 (PMC11649125; doi:10.1371/journal.pone.0315329)
Supplement: S1 Table — (DOCX) [file pone.0315329.s001.docx]

**S1 Table. AGB statistics for various grassland types in northern China and alpine grassland.**

| Grassaland type | Area (10^4^ km^2^) | Mean AGB (g/m^2^) |
| --- | --- | --- |
| temperate meadow steppe | 10.27 | 244.22±52.88 |
| temperate typical steppe | 54.30 | 172.96±186.26 |
| temperate desert steppe | 28.46 | 67.34±10.50 |
| apline steppe | 75.08 | 429.01±56.24 |
| tropical tussock | 0.06 | 810.16±122.80 |
| temperate meadow | 30.73 | 112.16±20.91 |
| apline meadow | 69.33 | 138.46±17.87 |
