# Supplementary material for: Spatiotemporal dynamics of grassland aboveground biomass in northern China and the alpine region: Impacts of climate change and human activities
Source: PLoS One. 2024 Dec 16;19(12):e0315329. doi: 10.1371/journal.pone.0315329 (PMC11649125; doi:10.1371/journal.pone.0315329)
Supplement: S2 Table — (DOCX) [file pone.0315329.s002.docx]

**S2 Table. The regression equations for grassaland AGB across different grassland types from 1981 to 2015.**

| Grassland type | Regression equation | r value | RMSE (g/m^2^) |
| --- | --- | --- | --- |
| Temperate meadow steppe | y = -0.262x + 768.026 | 0.050 | 54.418 |
| Temperate typical steppe | y = 0.061x + 50.512 | 0.017 | 37.896 |
| Temperate desert steppe | y = 0.101x -135.343 | 0.10 | 10.795 |
| Alpine steppe | y = 3.313x - 6192.58 | 0.606 | 45.182 |
| Tropical tussock | y = -2.965x + 6738.808 | 0.234 | 128.117 |
| Temperate meadow | y = 0.073x - 34.568 | 0.035 | 21.528 |
| Alpine meadow | y = 0.338x - 537.348 | 0.19 | 18.129 |
