# Supplementary material for: Spatiotemporal dynamics of grassland aboveground biomass in northern China and the alpine region: Impacts of climate change and human activities
Source: PLoS One. 2024 Dec 16;19(12):e0315329. doi: 10.1371/journal.pone.0315329 (PMC11649125; doi:10.1371/journal.pone.0315329)
Supplement: S3 Table — (DOCX) [file pone.0315329.s003.docx]

**S3 Table. The grassland AGB dynamics trends in northern China and alpine graslands.**

| Slope | Z value | AGB trend | Area percentage (%) |
| --- | --- | --- | --- |
| ≥ 0.0005 | ≥ 1.96 | Significant improved | 21.61 |
| ≥ 0.0005 | -1.96 – 1.96 | Slight improved | 18.99 |
| -0.0050 – 0.0005 | -1.96 – 1.96 | Stable | 19.36 |
| < -0.0005 | -1.96 – 1.96 | Slight degraded | 35.54 |
| < -0.0005 | < -1.96 | Significant degraded | 5.49 |
