# Supplementary material for: Spatiotemporal dynamics of grassland aboveground biomass in northern China and the alpine region: Impacts of climate change and human activities
Source: PLoS One. 2024 Dec 16;19(12):e0315329. doi: 10.1371/journal.pone.0315329 (PMC11649125; doi:10.1371/journal.pone.0315329)
Supplement: S4 Table — (DOCX) [file pone.0315329.s004.docx]

**S4 Table. The coefficient of variation (CV) of AGB in northern China and alpine graslands.**

| Coefficient of variation | Degree of variation | Percentage (%) |
| --- | --- | --- |
| C_v_≤0.05 | Low fluctuation change | 19.20 |
| 0.05<C_v_≤0.10 | Relatively low fluctuation change | 0.05 |
| 0.10<C_v_≤0.15 | Medium fluctuation change | 13.07 |
| 0.15<C_v_≤0.20 | Relatively high fluctuation change | 34.04 |
| C_v_>0.20 | High fluctuation change | 33.63 |
