# Supplementary material for: Spatiotemporal dynamics of grassland aboveground biomass in northern China and the alpine region: Impacts of climate change and human activities
Source: PLoS One. 2024 Dec 16;19(12):e0315329. doi: 10.1371/journal.pone.0315329 (PMC11649125; doi:10.1371/journal.pone.0315329)
Supplement: S5 Table — (DOCX) [file pone.0315329.s005.docx]

| **S5 Table. Correlation coefficients between AGB and temperature for different grassland types in different seasons.** | | | | |
| --- | --- | --- | --- | --- |
|  | Correlation coefficient | | | |
| Grassland Type | Spring | Summer | Autumn | Winter |
| Meadow steppe | 0.023 | -0.38 | -0.07 | 0.85 |
| Typical steppe | 0.06 | -0.10 | -0.12 | 0.83 |
| Desert steppe | 0.43 | 0.42 | 0.22 | 0.70 |
| Alpine steppe | 0.016 | -0.34 | 0.05 | 0.80 |
| Temperate meadow | -0.57 | -0.58 | -0.58 | 0.52 |
| Alpine meadow | 0.23 | 0.07 | 0.16 | 0.66 |
