# Supplementary material for: Spatiotemporal dynamics of grassland aboveground biomass in northern China and the alpine region: Impacts of climate change and human activities
Source: PLoS One. 2024 Dec 16;19(12):e0315329. doi: 10.1371/journal.pone.0315329 (PMC11649125; doi:10.1371/journal.pone.0315329)
Supplement: S6 Table — (DOCX) [file pone.0315329.s006.docx]

| **S6 Table. Correlation coefficients between AGB and precipitation for different grassland types in different seasons.** | | | | |
| --- | --- | --- | --- | --- |
|  | Correlation coefficient | | | |
| Grassland Type | Spring | Summer | Autumn | Winter |
| Meadow steppe | 0.36 | 0.46 | 0.32 | -0.16 |
| Typical steppe | 0.54 | 0.53 | 0.42 | -0.15 |
| Desert steppe | 0.68 | 0.58 | 0.61 | -0.15 |
| Alpine steppe | 0.40 | 0.52 | 0.50 | 0.17 |
| Temperate meadow | 0.79 | 0.75 | 0.79 | -0.21 |
| Alpine meadow | 0.68 | 0.30 | 0.72 | 0.09 |
