# Supplementary material for: Spatiotemporal dynamics of grassland aboveground biomass in northern China and the alpine region: Impacts of climate change and human activities
Source: PLoS One. 2024 Dec 16;19(12):e0315329. doi: 10.1371/journal.pone.0315329 (PMC11649125; doi:10.1371/journal.pone.0315329)
Supplement: S7 Table — (DOCX) [file pone.0315329.s007.docx]

| **S7 Table. Correlation coefficients between AGB and soil moisture for different grassland types in different seasons.** | | | | |
| --- | --- | --- | --- | --- |
|  | Correlation coefficient | | | |
| Grassland Type | Spring | Summer | Autumn | Winter |
| Meadow steppe | 0.51 | 0.47 | 0.39 | 0.33 |
| Typical steppe | 0.66 | 0.49 | 0.55 | 0.26 |
| Desert steppe | 0.67 | 0.53 | 0.67 | 0.08 |
| Alpine steppe | 0.40 | 0.30 | 0.29 | -0.06 |
| Temperate meadow | 0.80 | 0.83 | 0.83 | 0.01 |
| Alpine meadow | 0.42 | 0.23 | 0.34 | 0.03 |
