# Supplementary figures and images for: Spatiotemporal dynamics of grassland aboveground biomass in northern China and the alpine region: Impacts of climate change and human activities
Source: PLoS One. 2024 Dec 16;19(12):e0315329. doi: 10.1371/journal.pone.0315329 (PMC11649125; doi:10.1371/journal.pone.0315329)

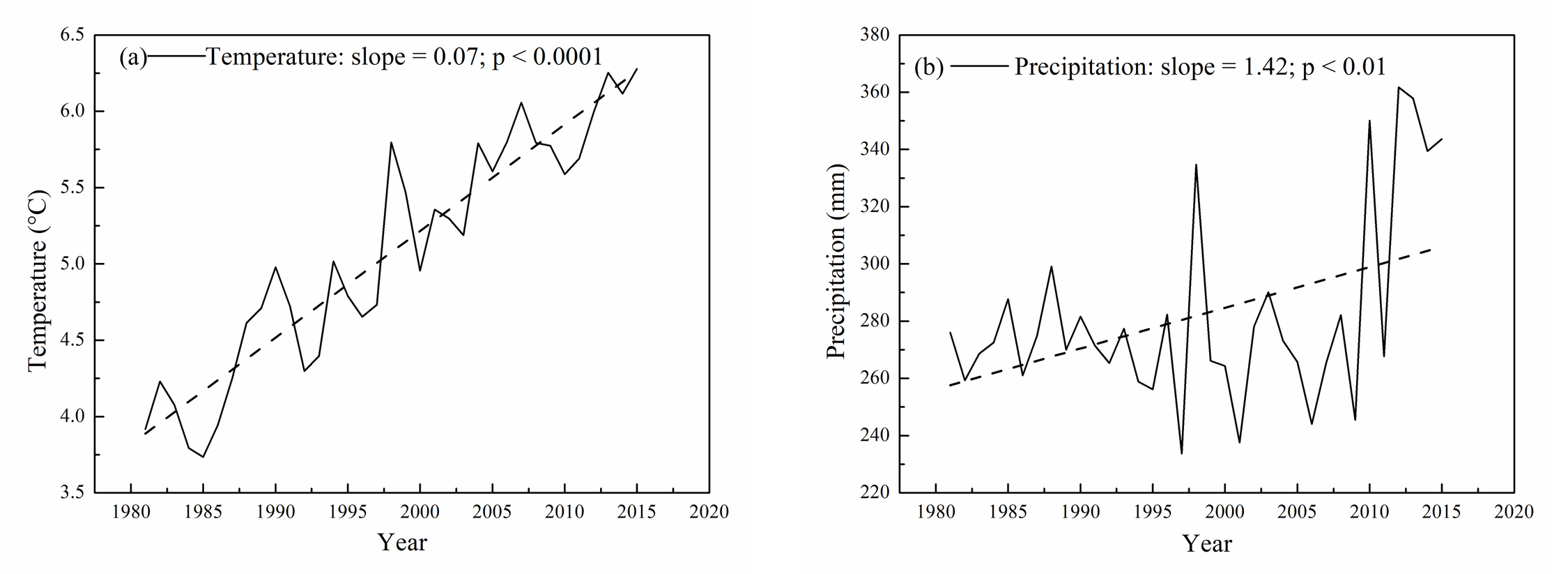

Supplement: S1 Fig — (TIF) [file pone.0315329.s008.tif]

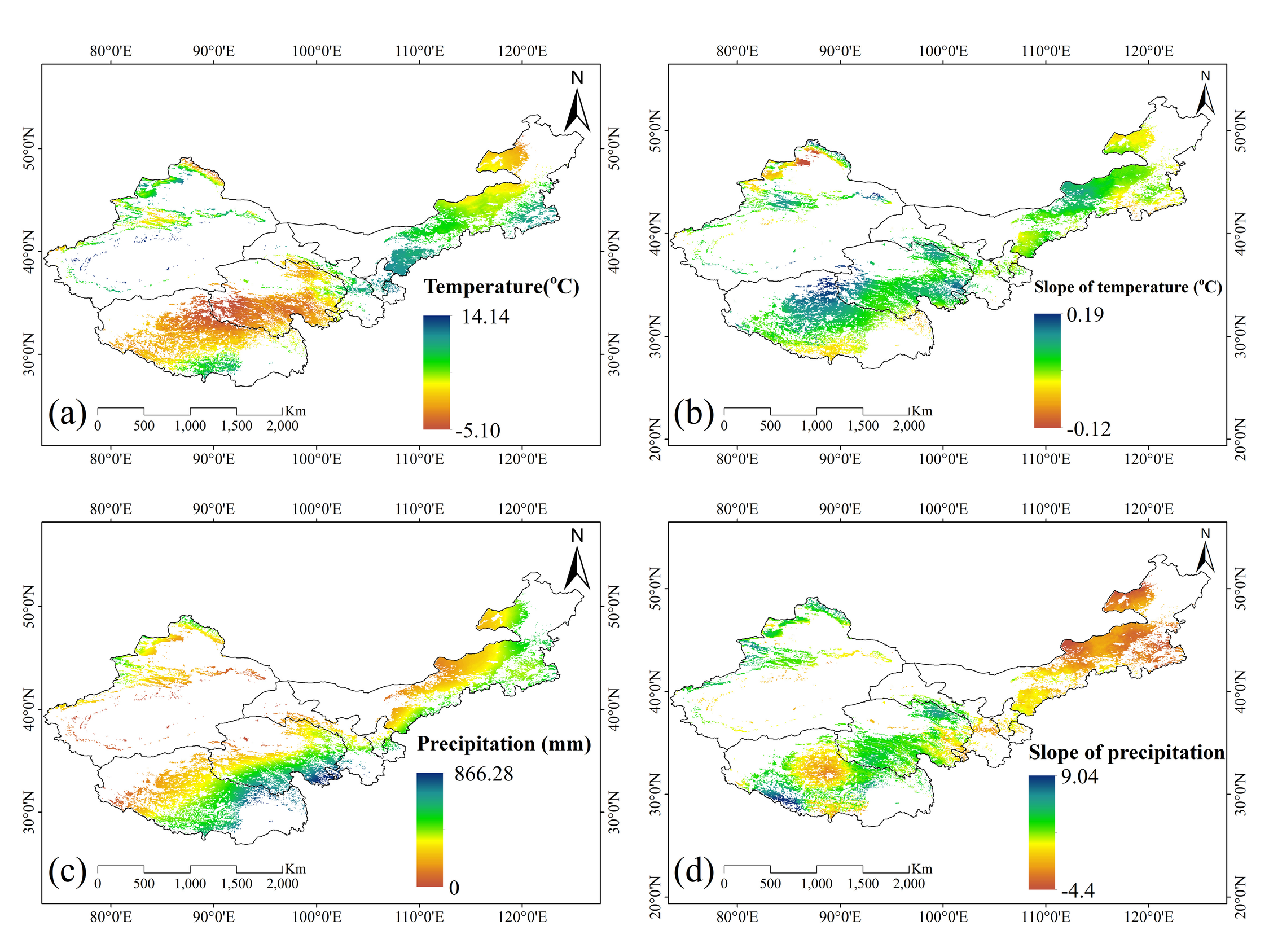

Supplement: S2 Fig — Source: Land use and cover data are from the Land Processes Distributed Active Archive Center (LP DAAC, https://lpdaac.usgs.gov).The study areas are from National Geomatics Center of China (https://www.ngcc.cn). (TIF) [file pone.0315329.s009.tif]
