# Supplementary material for: Spatiotemporal dynamics of grassland aboveground biomass in northern China and the alpine region: Impacts of climate change and human activities
Source: PLoS One. 2024 Dec 16;19(12):e0315329. doi: 10.1371/journal.pone.0315329 (PMC11649125; doi:10.1371/journal.pone.0315329)
Supplement: Attachment — Submitted filename: Response to Reviewers.docx [file pone.0315329.s010.docx]

**Reviewer #1:** Based on the NDVI data and meteorological data, this study estimated the AGB in northern China’s temperate and alpine grassland and its relationship with climatic variables. The results of this study may contribute to further understanding the relationships between AGB and climate change. However, there are some concerns that the authors should address before it can be accepted for publication.
**1. In the introduction, I suggest the authors add some contents about how environmental drivers influence the dynamics of grassland AGB.**

**Response**: We appreciate your suggestion to expand on how environmental drivers influence the dynamics of aboveground biomass (AGB). We have added a more detailed discussion in the introduction section to analyze the impacts of climatic factors, environmental conditions, and human activities on AGB dynamics. Additionally, we discuss the impacts of soil properties, nutrient availability, and soil moisture on vegetation growth and biomass accumulation, as well as human activities like grazing intensity can alter the structure and composition of plant communities, ultimately impacting AGB. We also explain the significance of studying northern grassland AGB and the impacts of driving factors on grassland AGB dynamics. We believed that these modifications enhance the manuscript and provide a clearer context for our study.
**2. In terms of the part two-materials and methods, it is better to simplify and integrate the part of data source and data analysis, as the classification may be inaccurate and the contents are a little prolix.**

**Response**: We have revised the “Materials and Methods” section to simplify and integrate the data source and data analysis parts, aiming for clearer organization and conciseness. This should enhance the readability and logical flow of the manuscript.
**3. Since data from 99 meteorological stations are used, it is better to add the distribution of meteorological stations.**

**Response**: Thank you for your valuable suggestion. We have taken the reviewer’s suggestion into consideration and have added the distribution of climate stations in Fig 1 as requested. This addition enhances the clarity of our study area and the data sources used.
**4. I suggest the authors add some contents on handling missing values and outliers in NDVI data and meteorological data.**

**Response**: We have added a detailed explanation of how we handled missing values and outliers in both the GIMMS NDVI and meteorological data in the manuscript. Missing values in NDVI data were addressed using linear interpolation. Savitzky-Golay filters was used to smooth gaps. Outlier in NDVI data were detected and removed using the interquartile range (IQR) method. For the meteorological data, we employed linear interpolation to handle missing values. Outlier were detected and addressed using the IQR method to ensure data integrity. We believe that these detailed explanations enhance the clarity of our data preprocessing procedure.

**5. In order to provide a comprehensive assessment of grassland AGB in Northern China, I suggest the authors add some time-scale analysis of grassland AGB.**

**Response**: We have added a new section analyzing the seasonal changes in grassland AGB. We have revised the structure: 3.2 Spatial distributions of annual and seasonal changes of grassland AGB; 3.2.1 Annual distribution of grassland AGB; 3.2.2 Seasonal distribution of grassland AGB. In new section, we analyzed the changes of grassland AGB across four seasons: spring, summer, autumn, and winter. We analyzed the various seasonal fluctuations, which may be influenced by variations in precipitation and temperature across season.
**6. When drawing pictures, pay attention to color brightness and saturation to give people a sense of comfort, the authors should modify the figure 5 and 6.**

**Response**: In the revised manuscript, figures 5 and 6 have been updated and are now labeled as Figs 6 and 7. These figures were created using ArcMap software. We have adjusted the color brightness and saturation to improve visual comfort.
**7. More mechanism explanations should be added to further explain the influence of driving factors on the dynamics of grassland vegetation.**

**Response**: We have made some revisions to address the suggestion for more mechanism explanations. We have added two new figure (Figs 7f and 7g) showing the relationship between AGB and soil moisture, grazing intensity. We relocated origin Fig 6f to Fig 8 for improved clarity.

In the revised manuscript, we provide a comprehensive analysis of the mechanism through which climate factors, such as temperature (T), precipitation (PPT), VPD, relative humidity (RH), solar radiation (SR), and soil moisture (SM), as well as human activities like grazing intensity impact on grassland AGB.

In Fig 8, we discuss the important roles that these climate factors and human activities play in promoting plant growth and biomass accumulation, thereby providing a deeper understanding of the dynamics of grassland.

In Fig 9, we added Figs 9f and 9g to analyze the relationship between AGB and soil moisture, as well as grazing intensity (GI). This addition allowed us to explore the mechanisms by which climate factors and soil moisture, grazing intensity impact grassland AGB, providing deeper insights into their interactions and effects on biomass dynamics.

These additions and adjustments enhance the mechanism explanation of how different factors affect grassland biomass.

**8. In order to further highlight the innovation of this article, it is better to compare the results of this study with some related studies.**

**Response**: We have revised the discussion section to include comparisons with related studies and to elaborate on the mechanism revealed by our research. We highlighted the responses of different grassland types to climate factors and human activities. This revision provides a clearer understanding of grassland dynamics and the complexities involved in managing these grassland resources effectively.
**9. In the uncertainty, I suggest the authors further discuss the uncertainty of remote sensing data including NDVI data (e.g., Shen et al., 2021; Wang et al., 2021) which may affect the research results.**
References:
Aboveground biomass and its spatial distribution pattern of herbaceous marsh vegetation in China. Science China Earth Sciences, 2021, 64: 1115-1125.
Spatiotemporal change of aboveground biomass and its response to climate change in marshes of the Tibetan Plateau. International Journal of Applied Earth Observation and Geoinformation, 2021, 102: 102385.

**Response:** In the revised manuscript, we have included a new discussion section titled “4.4 Assessing uncertainty in grassland AGB estimation.” In this section, we analyzed the uncertainties associated with NDVI and meteorological data, as well as the potential impacts of data preprocessing on our findings. This addition provides a clearer understanding of how these uncertainties affect the accuracy of grassland AGB estimations.
